# Pre- and post-COVID-19 all-cause mortality of Japanese citizens versus foreign residents living in Japan, 2015–2021

**DOI:** 10.1016/j.ssmph.2022.101114

**Published:** 2022-05-10

**Authors:** Cyrus Ghaznavi, Akifumi Eguchi, Yuta Tanoue, Daisuke Yoneoka, Takayuki Kawashima, Motoi Suzuki, Masahiro Hashizume, Shuhei Nomura

**Affiliations:** a Department of Health Policy and Management, School of Medicine, Keio University, Tokyo, Japan; b Medical Education Program, Washington University School of Medicine in St Louis, Saint Louis, USA; c Department of Global Health Policy, Graduate School of Medicine, The University of Tokyo, Tokyo, Japan; d Center for Preventive Medical Sciences, Chiba University, Chiba, Japan; e Institute for Business and Finance, Waseda University, Tokyo, Japan; f Tokyo Foundation for Policy Research, Tokyo, Japan; g Infectious Disease Surveillance Center, National Institute of Infectious Diseases, Tokyo, Japan; h Department of Mathematical and Computing Science, Tokyo Institute of Technology, Tokyo, Japan; i Center for Surveillance, Immunization, and Epidemiologic Research, National Institute of Infectious Diseases, Tokyo, Japan

**Keywords:** Japan, COVID-19, Foreign residents, Immigrants, All-cause mortality

## Abstract

Immigrants in Japan face multiple health care challenges. There is limited research addressing how all-cause mortality differs between foreign residents and Japanese citizens, including the impact of the COVID-19 pandemic. We assessed whether all-cause mortality rates between Japanese citizens and foreign residents living in Japan differ, and whether these differentials changed after the start of the COVID-19 pandemic. We conducted a cross-sectional analysis using vital statistical data of all deaths among citizens and foreign residents that occurred within Japanese borders aggregated every 6 months between January 1, 2015 and June 30, 2021. Data were used to calculate sex-, region-, and 20-year age group-specific standardized mortality rates using the direct method based on the population distribution of Japanese citizens in 2021 by sex, region, and 20-year age groups. Chi-squared tests and linear regression were used to assess whether the pandemic was associated with changes in mortality rates among groups and changes in the mortality differentials between citizens and non-citizens, respectively. All-cause mortality increased monotonically with age for men and women. Men had higher mortality than women, regardless of age or nationality. All-cause mortality is lower among immigrants than Japanese citizens between the ages of 20–59, but higher under the age of 20 and over the age of 59. The pandemic was associated with significant changes in mortality in most groups, but no statistically significant changes in the mortality differentials between immigrants and Japanese citizens were detected. Young immigrants are generally healthier than their Japanese counterparts, in line with the healthy migrant hypothesis. Younger migrants are at higher risk of mortality, possibly due to increased vulnerability to psychologic stress. Older migrant mortality converged with citizen mortality, consistent with acculturation that occurs with longer duration of residence. The pandemic did not exacerbate health inequities for foreign residents with respect to mortality.

## Highlights

• Foreign residents have lower mortality than citizens between the ages of 20–59.
• Foreign residents have higher mortality than citizens <20 and >59 years of age.
• COVID-19 did not change mortality differentials between immigrants and citizens.

## 1 Introduction

As of 2020, approximately 2.9 million foreign residents lived in Japan, comprising roughly 2.3% of the country's total population (e-Stat, 2020). Most immigrants hail from Asia, with China (778,000), Vietnam (448,000), Korea (427,000), and the Philippines (280,000) accounting for the top four exporters of foreign residents to Japan. The number of foreign residents in Japan has been generally increasing for several years (1.7 million in 2000 and 2.1 million in 2010), though it did decrease by approximately 50,000 between the end of 2019 and 2020 (e-Stat, 2000; 2010, 2019). Beginning in late January 2020, Japanese borders were closed to foreign nationals from a list of designated countries that grew to greater than 100 countries by May 2020 (Takahara, 2022). Hundreds of thousands of potential immigrants were unable to enter to enter the country during this period. Though borders were provisionally reopened to students, interns, and business travelers in November 2021, border closures were soon reinstated mere weeks later and extended until March 2022, at which point foreign nationals arriving for reasons other than tourism were allowed entry. Given that Japan's border closures were some of the strictest globally, the state of foreign residents in Japan has come under renewed attention.

In particular, there is significant concern that the COVID-19 pandemic will exacerbate already existing health inequities between foreign residents and citizens (Caron & Adegboye, 2021). Immigrants are more likely to experience COVID-19-related health disruptions because of crowded housing and large household size (Kjollesdal et al., 2022), difficulty with health literacy (Wang et al., 2020), concerns related to testing and treatment (e.g., requirements to present official identification or residency documentation) (Lechuga et al., 2022), language barriers (Caron & Adegboye, 2021), and inadequate insurance coverage (McFadden et al., 2022). Furthermore, a multi-country analysis has shown that the threat posed by COVID-19 has led to increased “othering” of immigrant communities and anti-immigrant sentiment (Mula et al., 2022). Taken together, citizenship status is a social determinant of health, and the “citizenship shield” functions as a protective factor with respect to accessing COVID-19-related health information, testing, care, vaccines, and financial support (Cadenas et al., 2022).

Japan's economy was also considerably impacted by the pandemic. Immigrant workers are particularly vulnerable to economic shocks, and the pandemic affected many of the sectors in which foreigners are concentrated: construction, food-production, hospitality, and healthcare, among others (Disney et al., 2022). COVID-19 triggered a 1.07 million drop in the number of non-regular workers between January and July 2020 (Abe, 2021); foreign workers were affected by widespread job losses that resonated throughout Japan (The Japan Times, 2021). Job losses, lack of telework flexibility, and housing insecurity disproportionately affect foreign workers and have profound effects on their health status (OECD, 2020; Solheim et al., 2022).

Historically, immigrants residing in Japan have faced challenges within the health care system, including language barriers and difficulty enrolling in universal health coverage (Yasukawa et al., 2019). Language barriers, in particular, have slowed Japan's vaccine response among the foreign resident community because the use of vaccine vouchers and reservation systems requires a certain degree of Japanese proficiency (Shirakawa, 2021). To address this barrier, some municipalities began translating the materials, but this led to foreign residents receiving vouchers after their citizen counterparts (Kyodo News, 2021). The recent hosting of the Tokyo 2020 Summer Olympics has shined a light on the importance of equipping the domestic health care system with tools to handle tourists and foreign residents who may not speak Japanese (Ono, 2020; Watanabe & Sakka, 2017).

Furthermore, some clinics may be reluctant to accept non-Japanese patients, particularly those without insurance; when they are accepted, they may be charged higher fees or discharged earlier than dictated by standard care (Kunii & Nomiyama, 1993; Yasukawa et al., 2019). Fear of stigmatization may lead some foreign residents to ignore illnesses for which they would otherwise pursue treatment (Doan et al., 2021; Kunii & Nomiyama, 1993). Psychological stress is also a major concern among immigrant populations: prior research has found that Korean, but not Chinese, immigrants have higher rates of suicide than their Japanese counterparts (Gilmour et al., 2019).

To the best of our knowledge, only one study – written in Japanese using 2010 summary data – has assessed immigrant all-cause mortality in Japan to date, finding that foreign resident mortality is less than that of their Japanese counterparts in younger age groups but switches to being greater among the elderly (Kobori et al., 2017). Furthermore, the effects of COVID-19 on health inequities between Japanese citizens and foreign residents has yet to be assessed despite concerning trends seen in other countries in which immigrants have disproportionately suffered from the pandemic (Indseth et al., 2021; Riley et al., 2021; Ross et al., 2020). Though several studies from Western countries have assessed these trends, there is a concerning dearth of research addressing this question among East Asian nations (Aldridge et al., 2018).

Given that the immigrant population is only expected to rise in Japan (Okada, 2018), further research is necessary. The objectives of the current study are twofold: first, we use national, individual-level mortality data to assess differences between all-cause mortality in Japanese citizens and foreign residents residing in Japan between 2015 and 2021. Second, we then test whether these trends and the mortality differentials between citizens and foreign residents were affected by the COVID-19 pandemic.

## 2 Material and methods

### 2.1 Data

We used daily and individual-level mortality data obtained from the vital statistics of the Ministry of Health, Labour and Welfare of Japan (MHLW) between January 1, 2015 and June 30, 2021. This data does not include those who stay in Japan for a short period of time without a residency card (*zairyūkādo*), such as for tourism, and only includes deaths that occurred within Japan's borders.

### 2.2 Calculation of mortality rates

To calculate crude mortality rates, the individual data was aggregated every six months and stratified by citizenship (Japanese citizen vs foreign resident), sex, age group (0–19, 20–39, 40–59, 60–79 and ≥ 80 years), and region (i.e., Hokkaido/Tohoku, Kanto, Chubu, Kinki, Chugoku/Shikoku, and Kyushu). Thus, the mortality rates in this study are not annual rates, as they are estimated for each half year. The number of Japanese citizens and foreign residents in any given year was obtained from population data in the Basic Resident Register (updated annually on January 1 of each year) (Ministry of Internal Affairs and Communications, 2021). The standardized mortality and the associated 95% confidence intervals stratified by sex, 20-year age group, and citizenship status were calculated via the direct standardization method, using the sex-, region-, and 20-year age group-specific Japanese population values from 2021 as the standard population distribution (Ministry of Internal Affairs and Communications, 2021).

### 2.3 Pre-vs post-COVID-19 analyses: changes in mortality trends

The COVID-19 pandemic was defined as beginning in January 2020 when the first official case was identified in Japan. The standardized mortality for any given sex, age, and citizenship group was compared before and after the COVID-19 pandemic using the chi-squared test; testing was performed by constructing a two-by-two table with pre-COVID-19 (aggregated from the first half of 2015 to the second half of 2019) and post-COVID-19 (aggregated from the first half of 2020 to the first half of 2021) mortality. The results of these tests are reported as ‘*p* (χ^2^)’ in Table 1. All p-values less than 0.05 were considered as statistically significant; p < 0.05 suggests that the mortality rate for any given sex, age, and citizenship group significantly changed (i.e., increased or decreased) post-January 2020 compared to pre-pandemic levels.

**Table 1:** Sex-, region-, and age-standardized mortality rates among foreign residents and Japanese citizens in Japan, 2015–2021, by sex, age group, and citizenship.

Age Group	Citizenship	Year	Half	Men			Women		
				Rate	Lower	Upper	Rate	Lower	Upper
0–19	Japanese	2015	1st	12.3	11.6	13.0	9.4	8.8	10.0
		2015	2nd	12.5	11.8	13.1	9.0	8.4	9.6
		2016	1st	11.7	11.1	12.4	9.2	8.6	9.8
		2016	2nd	11.6	11.0	12.3	9.0	8.4	9.6
		2017	1st	11.7	11.0	12.3	8.6	8.1	9.2
		2017	2nd	11.4	10.8	12.0	8.3	7.8	8.9
		2018	1st	10.9	10.3	11.5	9.0	8.4	9.5
		2018	2nd	11.4	10.8	12.1	8.8	8.2	9.4
		2019	1st	11.0	10.4	11.7	9.0	8.5	9.6
		2019	2nd	11.3	10.7	11.9	8.8	8.2	9.4
		2020	1st	10.4	9.8	11.0	8.1	7.6	8.7
		2020	2nd	10.8	10.2	11.4	8.3	7.7	8.9
		2021	1st	10.5	9.9	11.1	7.8	7.3	8.4
		*p* (χ^2^)		< 0.001			< 0.001		
	Foreign	2015	1st	23.0	22.1	23.9	12.3	11.6	12.9
		2015	2nd	12.6	11.9	13.3	15.7	14.9	16.4
		2016	1st	17.6	16.8	18.4	15.9	15.1	16.6
		2016	2nd	12.2	11.5	12.8	11.0	10.4	11.7
		2017	1st	14.5	13.8	15.3	8.0	7.5	8.6
		2017	2nd	13.0	12.4	13.7	9.8	9.2	10.4
		2018	1st	13.1	12.4	13.8	10.9	10.2	11.5
		2018	2nd	20.7	19.9	21.6	12.1	11.4	12.8
		2019	1st	16.0	15.3	16.8	13.0	12.3	13.7
		2019	2nd	17.3	16.5	18.1	9.3	8.7	9.9
		2020	1st	13.2	12.5	13.9	7.5	7.0	8.0
		2020	2nd	13.6	12.9	14.3	17.1	16.3	17.9
		2021	1st	15.6	14.8	16.3	4.8	4.4	5.3
		*p* (χ^2^)		< 0.001			< 0.001		
20–39	Japanese	2015	1st	31.7	30.8	32.7	16.2	15.5	16.9
		2015	2nd	29.8	28.8	30.7	16.4	15.7	17.1
		2016	1st	31.3	30.3	32.2	16.6	15.9	17.3
		2016	2nd	29.0	28.1	29.9	15.5	14.8	16.2
		2017	1st	29.8	28.9	30.8	15.5	14.8	16.1
		2017	2nd	28.6	27.7	29.6	14.7	14.0	15.4
		2018	1st	29.1	28.2	30.0	14.8	14.2	15.5
		2018	2nd	28.4	27.5	29.3	15.5	14.8	16.2
		2019	1st	29.1	28.2	30.1	15.0	14.4	15.7
		2019	2nd	26.9	26.0	27.8	15.4	14.7	16.1
		2020	1st	28.2	27.3	29.1	14.2	13.6	14.9
		2020	2nd	29.6	28.7	30.5	17.5	16.8	18.2
		2021	1st	29.6	28.7	30.5	16.8	16.1	17.5
		*p* (χ^2^)		0.404			0.009		
	Foreign	2015	1st	17.1	16.4	17.8	9.9	9.4	10.5
		2015	2nd	19.3	18.6	20.1	6.7	6.3	7.2
		2016	1st	12.0	11.4	12.6	8.1	7.6	8.6
		2016	2nd	15.8	15.1	16.5	7.7	7.2	8.1
		2017	1st	13.8	13.1	14.4	8.9	8.4	9.4
		2017	2nd	15.5	14.8	16.1	9.2	8.7	9.8
		2018	1st	14.6	13.9	15.2	6.6	6.2	7.1
		2018	2nd	14.1	13.5	14.7	5.7	5.3	6.1
		2019	1st	14.8	14.1	15.4	8.8	8.3	9.3
		2019	2nd	13.6	13.0	14.3	8.1	7.6	8.6
		2020	1st	10.5	9.9	11.0	6.3	5.9	6.8
		2020	2nd	17.2	16.5	17.9	8.2	7.7	8.7
		2021	1st	10.9	10.3	11.4	7.3	6.8	7.8
		*p* (χ^2^)		< 0.001			< 0.001		
40–59	Japanese	2015	1st	140.9	139.2	142.7	73.3	72.0	74.6
		2015	2nd	134.8	133.1	136.5	74.1	72.8	75.4
		2016	1st	138.6	136.8	140.3	74.7	73.4	76.0
		2016	2nd	130.3	128.6	132.0	72.3	71.1	73.6
		2017	1st	133.8	132.1	135.5	71.4	70.1	72.7
		2017	2nd	127.5	125.9	129.2	71.5	70.3	72.8
		2018	1st	130.5	128.8	132.2	71.8	70.6	73.1
		2018	2nd	126.6	125.0	128.3	71.5	70.2	72.8
		2019	1st	130.5	128.8	132.2	71.0	69.8	72.3
		2019	2nd	124.3	122.6	125.9	71.0	69.7	72.3
		2020	1st	127.4	125.7	129.1	70.2	68.9	71.4
		2020	2nd	129.2	127.5	130.9	72.3	71.0	73.6
		2021	1st	127.4	125.7	129.1	70.7	69.4	71.9
		*p* (χ^2^)		< 0.001			0.004		
	Foreign	2015	1st	120.4	118.8	122.0	69.3	68.0	70.5
		2015	2nd	110.2	108.6	111.7	63.5	62.3	64.7
		2016	1st	104.4	102.9	105.9	70.4	69.1	71.7
		2016	2nd	99.7	98.2	101.2	72.6	71.3	73.9
		2017	1st	99.5	98.1	101.0	53.7	52.6	54.8
		2017	2nd	97.2	95.7	98.7	65.1	63.8	66.3
		2018	1st	110.0	108.4	111.5	58.9	57.8	60.1
		2018	2nd	97.7	96.3	99.2	57.3	56.1	58.4
		2019	1st	107.2	105.7	108.7	59.3	58.1	60.4
		2019	2nd	93.1	91.7	94.5	56.0	54.8	57.1
		2020	1st	102.9	101.4	104.4	64.0	62.8	65.2
		2020	2nd	112.5	111.0	114.1	56.9	55.7	58.0
		2021	1st	104.7	103.2	106.2	54.5	53.4	55.6
		*p* (χ^2^)		< 0.001			< 0.001		
60–79	Japanese	2015	1st	913.5	908.7	918.3	416.0	412.9	419.1
		2015	2nd	876.1	871.4	880.8	397.1	394.1	400.1
		2016	1st	898.4	893.6	903.2	406.6	403.6	409.7
		2016	2nd	869.8	865.1	874.5	393.9	390.9	396.9
		2017	1st	905.6	900.8	910.4	405.0	401.9	408.0
		2017	2nd	870.2	865.5	874.9	389.6	386.6	392.6
		2018	1st	908.4	903.6	913.2	407.8	404.7	410.9
		2018	2nd	875.2	870.5	879.9	395.7	392.7	398.7
		2019	1st	905.9	901.1	910.7	405.3	402.2	408.4
		2019	2nd	884.1	879.3	888.8	395.5	392.4	398.5
		2020	1st	891.7	886.9	896.4	393.6	390.6	396.6
		2020	2nd	887.7	882.9	892.4	397.4	394.3	400.4
		2021	1st	902.2	897.4	907.0	405.0	401.9	408.0
		*p* (χ^2^)		0.050			0.011		
	Foreign	2015	1st	1012.5	1007.4	1017.6	439.5	436.3	442.7
		2015	2nd	922.2	917.3	927.0	438.9	435.7	442.1
		2016	1st	963.5	958.5	968.4	447.6	444.4	450.8
		2016	2nd	901.4	896.6	906.1	415.7	412.5	418.8
		2017	1st	892.9	888.2	897.7	423.3	420.1	426.4
		2017	2nd	963.1	958.1	968.0	465.3	462.0	468.6
		2018	1st	876.8	872.1	881.5	469.1	465.8	472.4
		2018	2nd	944.7	939.9	949.6	407.2	404.1	410.3
		2019	1st	892.4	887.7	897.2	422.8	419.6	425.9
		2019	2nd	919.7	914.9	924.6	426.7	423.6	429.9
		2020	1st	985.6	980.6	990.6	410.1	407.0	413.1
		2020	2nd	902.6	897.8	907.4	399.8	396.8	402.9
		2021	1st	938.9	934.0	943.8	376.7	373.7	379.7
		*p* (χ^2^)		< 0.001			< 0.001		
≥ 80	Japanese	2015	1st	5227.0	5205.5	5248.4	3775.8	3761.9	3789.6
		2015	2nd	4975.5	4954.5	4996.5	3588.9	3575.4	3602.5
		2016	1st	5053.8	5032.7	5075.0	3649.2	3635.6	3662.9
		2016	2nd	5027.7	5006.6	5048.8	3643.1	3629.5	3656.7
		2017	1st	5149.6	5128.3	5171.0	3756.3	3742.5	3770.1
		2017	2nd	4974.1	4953.2	4995.1	3603.6	3590.1	3617.1
		2018	1st	5090.3	5069.1	5111.5	3734.3	3720.5	3748.1
		2018	2nd	4856.1	4835.3	4876.8	3582.6	3569.1	3596.1
		2019	1st	5023.2	5002.1	5044.2	3721.6	3707.8	3735.4
		2019	2nd	4867.7	4847.0	4888.5	3625.4	3611.8	3638.9
		2020	1st	4811.9	4791.3	4832.6	3559.9	3546.5	3573.4
		2020	2nd	4863.8	4843.0	4884.5	3578.9	3565.4	3592.3
		2021	1st	4986.8	4965.8	5007.8	3695.4	3681.7	3709.1
		*p* (χ^2^)		< 0.001			< 0.001		
	Foreign	2015	1st	6438.0	6414.3	6461.7	3983.1	3968.9	3997.3
		2015	2nd	5867.5	5844.8	5890.2	3852.4	3838.4	3866.4
		2016	1st	5318.0	5296.4	5339.7	3972.0	3957.9	3986.2
		2016	2nd	5894.5	5871.8	5917.2	3818.3	3804.3	3832.2
		2017	1st	5719.6	5697.2	5742.0	4019.9	4005.6	4034.2
		2017	2nd	5474.8	5452.9	5496.8	3860.7	3846.7	3874.7
		2018	1st	5673.5	5651.1	5695.8	4038.5	4024.2	4052.8
		2018	2nd	5464.4	5442.4	5486.3	3880.6	3866.6	3894.6
		2019	1st	5113.6	5092.3	5134.8	3874.5	3860.4	3888.5
		2019	2nd	5444.2	5422.3	5466.1	3919.3	3905.2	3933.4
		2020	1st	5254.6	5233.0	5276.1	3704.5	3690.8	3718.2
		2020	2nd	5409.9	5388.0	5431.7	4007.8	3993.6	4022.1
		2021	1st	5606.3	5584.1	5628.5	4049.5	4035.2	4063.8
		*p* (χ^2^)		< 0.001			0.784		

Half = 1st (January to June) vs 2nd (July to December).

Lower = lower bound of standard error; Upper = upper bound of standard error.

Rates shown are mortality per 100,000 population.

*p* for trend refers to the *p* value associated with the chi-squared analysis.

*p* (interaction) refers to the *p* value associated with the coefficient for the interaction.

### 2.4 Pre-vs post-COVID-19 analyses: changes in mortality differentials between Japanese citizens and foreign residents

**Fig. 1:**
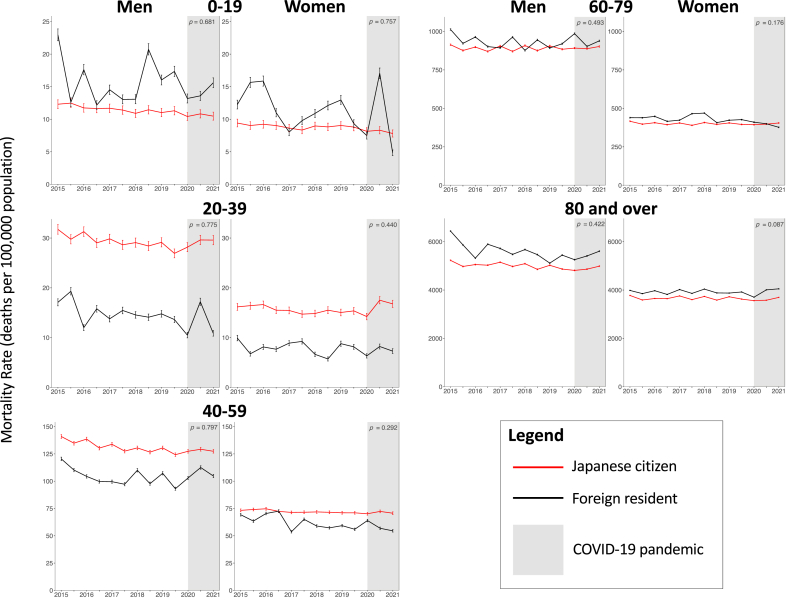
Sex-, region-, and age-standardized mortality rates among foreign residents and Japanese citizens in Japan, 2015–2021, by sex, age group, and citizenship. Fig. 1 Legend: Bars display standard error. P-values correspond to the significance testing for changes in the mortality differential between citizens and foreign residents pre- and post-pandemic (i.e., p-value for interaction term).

To check whether the difference in mortality between Japanese citizens and foreign residents within a given sex- and age-stratified group changed significantly during the COVID-19 pandemic, we conducted linear regression with the outcome variable defined as the difference between citizen and foreigner mortality rates. The following covariates were included in the model: time, a COVID-19 dummy variable (pre-vs post-January 2020), and their interaction term. Time was encoded as a variable such that the first half of 2015 was set to 2015.0, the second half was set to 2015.5, and so on until the first half of 2021 (2021.0). The dummy variable was defined as 0 pre-COVID-19 (all time points from the first half of 2015 to the second half of 2019) and defined as 1 for time points after the start of the pandemic (the first half of 2020 to the first half of 2021). The statistical significance of the interaction term, which was tested with t-tests, was used to determine whether there was a change in the mortality differential between the two groups before and after the start of the pandemic. The results of these tests (p-values of the interaction term) are shown in Fig. 1. All p-values less than 0.05 were considered as statistically significant. Significance suggests that the mortality differential between citizens and foreign residents for any given sex and age group changed after the start of the pandemic.

## 3 Results

### 3.1 Mortality rates

Standardized mortality rates stratified by sex and age group are shown in Fig. 1 and Table 1. For both men and women, mortality increases monotonically with age group. For any given age group, men have higher mortality than women. Compared to Japanese citizens, immigrants generally have higher mortality in the youngest age group (0–19 years), lower mortality between 20 and 59 years, and slightly higher mortality above the age of 59. The mortality differential is highest in the 20–39 year age group.

### 3.2 Pre-vs post-COVID-19 analyses

The results of the chi-squared analyses for changes in mortality trends are shown in Table 1, and the regression testing for changes in the mortality differential before and after the start of the COVID-19 pandemic are shown in Fig. 1. Significant changes in mortality pre-vs post-pandemic were found in all sex, age, and citizenship groups with the exception of 20–39 and 60-79 year-old Japanese males and foreign resident females above the age of 80 years. No significant changes in the mortality differentials between citizens and foreign residents pre-vs post-pandemic were detected for men or women in any age group.

## 4 Discussion

Using national Japanese mortality data, we assessed trends in mortality between 2015 and 2021 in Japanese citizens and foreign residents. We found that among those aged 19 or less and 60 and over, immigrants had higher mortality than their Japanese counterparts, but that these trends reversed among those aged 20–59 years. Furthermore, our results suggest that though mortality rates changed after the start of the pandemic, the mortality differentials between foreign residents and Japanese citizens did not change significantly.

That immigrants appear to have lower mortality rates than their Japanese counterparts between the ages of 20 and 59 is likely a manifestation of the *healthy migrant effect*, in which those who migrate represent a healthier subpopulation of their native country (Aldridge et al., 2018). The process of migrating and subsequently working in the host country necessarily self-selects for those who are healthy enough to emigrate and participate in the workforce. Notably, research from the US has shown that as immigrants remain abroad for longer periods of time, their health status and risk factors begin to resemble that of the native citizen population, a process known as *unhealthy assimilation* (Antecol & Bedard, 2006; Singh & Siahpush, 2002). As length of stay increases, the process of acculturation begins to overtake that of the healthy migrant effect, and immigrant mortality converges with citizen mortality, though it typically does not overtake citizen rates as has been seen in Canada and Norway (Omariba et al., 2014; Syse et al., 2016). Our findings show the convergence of foreign resident and Japanese citizen mortality with increasing age; furthermore, we find that immigrant mortality overtakes citizen mortality in the highest age groups, albeit only slightly. Though the reasons for this reversal are unclear, it is possible that the effects of unhealthy assimilation are stronger in Japan than other countries because of systemic biases in the health care system, such as language barriers, insurance enrolment difficulties, and fear of stigmatization in clinical spaces (Kunii & Nomiyama, 1993; Yasukawa et al., 2019).

Notably, foreign residents were found to have slightly higher mortality in the youngest age group in our analyses, seemingly in direct opposition to the healthy migrant effect. Research in Sweden has recapitulated this trend (Juarez et al., 2018), finding that migrants younger than 18 years comprise a vulnerable population, mainly due to psychological stress. Indeed, young Koreans living in Japan have higher suicide mortality than their Japanese counterparts (Gilmour et al., 2019). The pandemic likely disproportionately affected the mental health of immigrants: increased anxiety due to COVID-19 has been noted among Chinese women living in Japan (Luo & Sato, 2021), international students in China (Fakhar et al., 2020), and immigrants in South Korea (Acharya et al., 2021). Furthermore, research in Japan has found that infant mortality and stillbirth rates are significantly higher for immigrant women than their Japanese counterparts, in part due to language barriers, relatively low socioeconomic status, and underutilization of health care services (Kita et al., 2015). Notably, reasons for migration in this age group differ from older age groups in that employment is much less common; instead, many young immigrants may have been born in Japan (such as the Korean *zainichi* population), come with their parents, or participate in internships and educational opportunities. Children, in particular, may suffer if their parents are not able to take full advantage of local health care/insurance systems, do not have stable employment, or are limited in providing safe housing and food security (Cadenas et al., 2022; Caron & Adegboye, 2021; OECD, 2020).

There is a significant body of literature describing the difficulties that foreign residents in Japan face with respect to accessing and using health care, including language barriers, lack of access to health-related information, stigmatization, and difficulties in enrolling into the national insurance system (Kita et al., 2015; Kunii & Nomiyama, 1993; Yasukawa et al., 2019). The need for improved medical interpretation when handling non-Japanese speaking patients has long been recognized in the setting of a growing foreign resident population, especially in less urban areas (Kawaguchi & Ogasawara, 2015; Kishimoto & Noda, 2016; Ono, 2020). Prior research regarding Latin American immigrants in Japan found uninsurance rates up to 20%, compared to 1.3% for the general population (Suguimoto et al., 2012). Furthermore, immigrants may suffer from various socioeconomic disparities that predispose them to poorer health outcomes, such as low economic status, unstable employment, poor housing, and food insecurity (Caron & Adegboye, 2021; Doan et al., 2021; Kita et al., 2015). Unemployed and student foreign residents have been identified as high-risk groups with respect to health care access (Higuchi et al., 2021), as are those who have been residing in Japan for shorter periods of time (Shakya et al., 2018). However, there is some evidence to suggest that there are no significant differences in mortality or length of stay between Japanese citizens and foreign residents at tertiary care centers in Japan, though the research is limited in scope (Ishii et al., 2020).

The COVID-19 pandemic has ignited global concern for marginalized populations, particularly immigrants and refugees. The pandemic has widened already existing health inequities (Caron & Adegboye, 2021). COVID-19 has been found to have both higher infection rates and hospitalization rates among immigrants in Norway (Indseth et al., 2021); low socioeconomic status and crowded housing were found to be determinants for this trend (Kjollesdal et al., 2022). However, the effects on all-cause mortality have been mixed: research in Sweden has found that immigrants from low- and middle-income countries had a higher risk of death from COVID-19 but not for all-causes (Drefahl et al., 2020), and an Italian study found that immigrants and native Italians suffered from similar COVID-19 mortality, with the exception of higher mortality observed specifically among those from Latin America (Giacomelli et al., 2022). Contrastingly, migrants in England experienced a higher relative increase in all-cause mortality than their citizen counterparts (Public Health England, 2020), as did Latino immigrants in California (Riley et al., 2021). With respect to infection alone, Mexican immigrants in the US (Vilar-Compte et al., 2022) and ethnic minorities in the Netherlands suffered from higher rates of COVID-19 infection (Coyer et al., 2022), and low health literacy was found to be a predictor of this trend. Research from Canada has found higher rates of vaccine hesitancy and skepticism among immigrant communities, which would translate to higher rates of infection, severe illness, and mortality due to COVID-19 (Lin, 2022). In contrast to many prior studies, our findings suggest that the mortality differential between immigrants and Japanese citizens remained stable during the pandemic. The lack of changes during the pandemic period is likely attributable to Japan's success in controlling the pandemic, relatively low case counts, and minimal mortality compared to the global average (McCurry, 2021).

This study has limitations. First, we did not have access to data regarding country of origin or duration of residence in Japan. Thus, though older age groups included in our analysis are likely comprised of many residents who have resided in Japan long-term, we cannot make definitive claims regarding the extent of acculturation among the group as a whole. Further studies considering these variables are warranted. Second, it is possible that during the pandemic, immigrants who were ill or concerned about passing in the near future may have returned to their home country, a phenomenon known as *salmon bias* (Di Napoli et al., 2021). Foreign resident registration data suggests that the number of immigrants living in Japan slightly decreased by approximately 50,000 (1.6%) after the start of the pandemic (e-Stat, 2019; 2020), suggesting that the potential impact of salmon bias would be minimal. However, these values do not reflect those who voluntarily left Japan despite remaining validity of their foreign resident status. If salmon bias were at play in Japan, it would have artificially lowered immigrant mortality rates, particularly in older age groups, and thus may have masked a potential mortality disparity between these two groups.

## 5 Conclusion

In this study using national Japanese vital statistical data, we found that foreign residents have lower mortality than their citizen counterparts between the ages of 20 and 59, consistent with the healthy migrant hypothesis. That immigrants younger than 20 years may suffer from higher mortality rates may reflect increased vulnerability to psychologic stress and parental socioeconomic factors. Similarly, immigrants above the age of 59 years may suffer from unhealthy assimilation and find difficulty overcoming systemic biases in the healthcare system, such as language barriers, insurance difficulties, and stigmatization in clinical spaces. We also found that the COVID-19 pandemic did not exacerbate mortality differentials between citizens and foreign residents. Further research regarding health-seeking behaviors and the treatment of immigrants in the Japanese health care system is warranted. Given that Japan's immigrant population is only expected to increase moving forward, the health of foreign residents and the social disparities that underlie their health outcomes also merit further attention.

## Contributors

Conception/design of the work: CG, AE, SN; analysis of data: AE, YT, DY; interpretation of findings: all authors; drafting of the work: CG, DY; substantially revised the work: all authors.

## Data sharing

The mortality data have been obtained through a restricted data-use agreement with the Ministry of Health, Labour and Welfare, Japan, and are therefore not available for public dissemination.

## Funding

The present work was supported in part by a grant from the 10.13039/501100003478Ministry of Health, Labour and Welfare of Japan (20HA2007), and the 10.13039/501100001700Ministry of Education, Culture, Sports, Science and Technology of Japan (21H03203). The funding sources had no role in the study design, data collection, data analysis, data interpretation or preparation of the manuscript.

## Ethics statement

Ethical approval was granted by the ethics committee of the National Institute of Infectious Diseases, under authorization number 1174.

## Author statement

Cyrus Ghaznavi: Conceptualization; Methodology; Software; Validation; Formal analysis; Investigation; Writing - Original Draft; Writing - Review & Editing; Visualization. Akifumi Eguchi: Conceptualization; Methodology; Software; Validation; Formal analysis; Investigation; Writing - Original Draft; Writing - Review & Editing. Yuta Tanoue: Conceptualization; Methodology; Software; Validation; Formal analysis; Investigation; Writing - Original Draft; Writing - Review & Editing. Daisuke Yoneoka: Conceptualization; Methodology; Software; Validation; Formal analysis; Investigation; Writing - Original Draft; Writing - Review & Editing. Takayuki Kawashima: Conceptualization; Methodology; Software; Validation; Formal analysis; Investigation; Writing - Original Draft; Writing - Review & Editing. Motoi Suzuki: Resources; Data Curation; Writing - Original Draft; Writing - Review & Editing; Visualization; Supervision; Project administration; Funding acquisition. Masahiro Hashizume: Resources; Data Curation; Writing - Original Draft; Writing - Review & Editing; Visualization; Supervision; Project administration; Funding acquisition. Shuhei Nomura: Conceptualization; Methodology; Software; Validation; Formal analysis; Investigation; Resources; Data Curation; Writing - Original Draft; Writing - Review & Editing; Visualization; Supervision; Project administration; Funding acquisition.

## Declaration of competing interest

The authors declare that they have no competing interests or financial disclosures.
